# Hot Water for the Heart

**Published:** 1881-11

**Authors:** 


### HOT WATER FOR THE HEART.

In a letter to the *Lancet*, Dr. A. Paggi records the following observation: He states that in Paris he saw a case in which, under the inhalation of chloroform, the heart ceased to beat, and artificial respiration for ten minutes failed to restore circulation, when Dr. Labbe dipped a large cloth in boiling water and applied it to the region of the heart, with the result of immediately restoring the action of that organ.

